# Transient Receptor Potential Ankyrin Type-1 Channels as a Potential Target for the Treatment of Cardiovascular Diseases

**DOI:** 10.3389/fphys.2020.00836

**Published:** 2020-07-30

**Authors:** Song Gao, Keneilwe Kenny Kaudimba, Shanshan Guo, Shuang Zhang, Tiemin Liu, Peijie Chen, Ru Wang

**Affiliations:** ^1^School of Kinesiology, Shanghai University of Sport, Shanghai, China; ^2^Institute of Sport Science, Harbin Sport University, Harbin, China; ^3^State Key Laboratory of Genetic Engineering, Institute of Metabolism and Integrative Biology, Human Phenome Institute, Department of Endocrinology and Metabolism, and School of Life Sciences, Zhongshan Hospital, Fudan University, Shanghai, China

**Keywords:** TRPA1 channel, vascular physiology, atherosclerosis, heart failure, myocardial fibrosis, arrhythmia

## Abstract

Cardiovascular disease is one of the chronic conditions with the highest mortality rate in the world. Underlying conditions such as hypertension, metabolic disorders, and habits like smoking are contributors to the manifestation of cardiovascular diseases. The treatment of cardiovascular diseases is inseparable from the development of drugs. Consequently, this has led to many researchers to focus on the search for effective drug targets. The transient receptor potential channel Ankyrin 1 (TRPA1) subtype is a non-selective cation channel, which belongs to the transient receptor potential (TRP) ion channel. Previous studies have shown that members of the TRP family contribute significantly to cardiovascular disease. However, many researchers have not explored the role of TRPA1 as a potential target for the treatment of cardiovascular diseases. Furthermore, recent studies revealed that TRPA1 is commonly expressed in the vascular endothelium. The endothelium is linked to the causes of some cardiovascular diseases, such as atherosclerosis, myocardial fibrosis, heart failure, and arrhythmia. The activation of TRPA1 has a positive effect on atherosclerosis, but it has a negative effect on other cardiovascular diseases such as myocardial fibrosis and heart failure. This review introduces the structural and functional characteristics of TRPA1 and its importance on vascular physiology and common cardiovascular diseases. Moreover, this review summarizes some evidence that TRPA1 is correlated to cardiovascular disease risk factors.

## Introduction

At present, the incidence of cardiovascular disease has increased significantly and has become a significant health issue of global concern (North and Sinclair, 2012; Alali et al., 2017). The rapid world economic growth has aggravated the double pressure of work coupled with unhealthy lifestyles, which increases the chance of being exposed to independent risk factors (Luan et al., 2019). Independent risk factors for cardiovascular diseases include smoking, diabetes, hypertension, dyslipidemia, and obesity (Guo et al., 2020; Wang R. et al., 2020). Long-term exposure to risk factors exacerbates the impacts of cardiovascular disease (Pei et al., 2014; Wu et al., 2019a). Common cardiovascular diseases include atherosclerosis, myocardial fibrosis, heart failure, and arrhythmia. Almost all cardiovascular diseases require long-term medication to correct their physiological and pathological changes (Huddy et al., 2013). However, the side effects of long-term medication should be addressed aggressively (Ramkumar et al., 2016). Treating arrhythmia with some antiarrhythmic drugs can cause bradycardia and atrioventricular block (Sardar et al., 2016). Furthermore, long-term use of cardiotonic drugs can cause arrhythmia in addition to gastrointestinal symptoms (Ward et al., 1983; Hamlin, 2007; Sala and Bellin, 2017; Ivanov and Lagunin, 2019). Therefore, it is particularly important to explore new drug targets for the treatment of cardiovascular diseases. The discovery of drug targets is the basis of new drug development. The discovery of a new drug target is a breakthrough in the development of a series of new drugs. Currently, the targets of cardiovascular diseases are updated constantly. Researchers started with the pathogenesis of cardiovascular diseases looking for key receptors, ion channels, and signaling pathways that mediate the formation of cardiovascular diseases as targets for drug development (Overington et al., 2006; Maclay and MacNee, 2013; Mokou et al., 2017). Common drug targets for cardiovascular diseases are Rho kinase (Shimokawa et al., 2016), M3 receptor in functional muscarinic acetylcholine receptor (Kovacevic et al., 2015; Rhoden et al., 2019), S1P signaling pathway (sphingosine 1-phosphate) (Yung et al., 2017), and miR-145 (Massy et al., 2017; Sahu et al., 2017).

Transient receptor potential (TRP) channels are widely regarded as drug targets (Moran, 2018). TRP was first discovered when studying the visual conduction system of *Drosophila melanogaster* (Katz et al., 2013). In subsequent studies, more than 50 channel members of this family have been found from yeast, insects, fish, and mammals, of which more than 30 were found in mammals (Moran et al., 2004; Vriens et al., 2004; Nilius and Owsianik, 2011). Based on the differences in amino acid sequence homology of 33 TRP channels found in mammals, the TPR channels are divided into seven subfamilies, namely, TRPA, TRPC, TRPV, TRPM, TRPP, TRPML, and TRPN (Ramsey et al., 2006). Previous research has shown that members of the TRP family closely relate to cardiovascular disease (Ma et al., 2017). TRPV1 channel can regulate vascular smooth muscle and improve hypertension, causing heart dysfunction under cold exposure (Zhang et al., 2012; Shanks et al., 2019). The TRPV1 and TRPV4 channels are involved in the cardiovascular protection of hypoxia. TRPM7 is significantly upregulated in atrial fibroblasts from patients with atrial fibrillation (Yue et al., 2015). However, the role of TRPA1 in cardiovascular diseases is uncertain. In recent years, research on TRPA1 has been increasing, hence a close relationship between TRPA1 and cardiovascular diseases is established (Bodkin and Brain, 2011). Atherosclerosis causes chronic inflammation, and the activation of TRPA1 suppresses this inflammation (Bautista et al., 2006; Bonet et al., 2013). Moreover, some studies found that TRPA1 is also involved in the process of oxidative stress and myocardial fibrosis (Nilius and Szallasi, 2014; Wang et al., 2018).

In this review, researchers summarized the structure and the functions of TRPA1, which commonly uses agonists and inhibitors. Furthermore, the review summarizes the potential role of TRPA1 in regulating the pathophysiology of the cardiovascular system, including vascular physiology, atherosclerosis, myocardial fibrosis, heart failure, and arrhythmia (Table 1). Finally, this paper presents some evidence that TRPA1 is closely linked to cardiovascular disease risk factors.

**TABLE 1:** Role of the TRPA1 channel in the cardiovascular system.

**Diseases**	**Animals**	**Expression in heart and vasculature**	**Effects**	**Mechanisms**	**References**
Vascular physiology	Female CD1, C57BL/6, CGRP-/-, TRPV1-/-, and TRPA1-/- mice	–	4-ONE (1–30 nmol, intraplantar injection) triggers a vasodilation response, but not in TRPA1-/- mice	TRPA1-dependent neurogenic vasodilatation	Graepel et al., 2011
	Male Sprague–Dawley rats	Trigeminal root ganglia neurons	AITC (100 μM, intranasal administration) and acrolein (30 μM, intranasal administration) increase cerebral blood flow, but the effect is blocked by HC-030031 (50 μM, intranasal administration)	Neurogenic vasodilation	Kunkler et al., 2011
	Male CD1, CGRP-/-, TRPV1-/-, and TRPA1-/- mice (8–12 weeks old)	–	Cinnamaldehyde (1–30%) increases the blood flow, but not in HC-030031 (100 mg/kg)-treated and TRPA1 knockout mice	Neurogenic vasodilation	Aubdool et al., 2016
	Male Sprague–Dawley rats	Endothelial cells	AITC-induced (3–100 μM) cerebral artery dilation was abolished by the administration of HC-030031 (3 μM)	Endothelium-dependent vasodilation	Earley et al., 2009
	Adolescent rats	Endothelial cells	AITC (15–60 μM) evokes graded cerebral artery vasodilation	Endothelium-dependent vasodilation	Qian et al., 2013
	CD1, CGRP-/-, TRPV1-/-, and TRPA1-/- mice	–	Cinnamaldehyde (80–320 μM/kg) induces a transient hypotensive response followed by a sustained hypertensive response	Autonomic system reflexes	Pozsgai et al., 2010
Atherosclerosis	Male C57BL/6, apoE-/- and apoE-/-TRPA1-/- mice (8 weeks old)	Macrophages	AITC (10 mg/kg/day, 4 weeks, i.g.) suppresses atherosclerosis; HC-030031 (10 mg/kg/day, 4 weeks, i.g.) and TRPA1 knockout exacerbate atherosclerosis	Cholesterol metabolism and inflammation in macrophages	Zhao et al., 2016
	Male C57BL/6, apoE-/-, and apoE-/-TRPA1-/-mice (6–8 weeks old)	Macrophages	TRPA1-/-ApoE-/- mice showed a significant increase in atherosclerosis plaques; activation of TRPA1 by CIN sharply reduced atherosclerosis progression	Inflammation in macrophages	Wang Q. et al., 2020
Myocardial fibrosis	Male C57BL/6 mice (8–10 weeks old)	Cardiomyocytes and macrophages	HC-030031 (10 mg/kg/day, 4 weeks, i.g.) and TCS-5861528 (3 mg/kg/day, 4 weeks, i.g.) ameliorate cardiac hypertrophy and heart failure	Inhibits Ca^2+^-dependent signal pathways and macrophage polarization	Wang et al., 2018
	–	Human adult ventricular cardiac fibroblasts	HC-030031 (100 μM) and siRNA targeting the TRPA1 channel inhibit methylglyoxal-induced (300 μM) proliferation of cardiac fibroblasts	Inhibits Ca^2+^ entry	Oguri et al., 2014
	–	Cardiac fibroblasts	Activating TRPA1 with a specific agonist AITC promoted the synthesis and secretion of CGRP, as well as intracellular Ca^2+^	Increasing autocrine CGRP by activating TRPA1 can ameliorate cardiac fibrosis	Li et al., 2019
Heart failure	Male SD rats	Cardiomyocytes and dorsal root ganglia cell	The measured physiological response to topical application of AITC to both the lung and heart surface was blunted	*In vivo* reduction of TRPA1 expression was, in part, caused by CHF-related tissue ischemia and inflammation	Adam et al., 2019
Arrhythmia	Female B6129 mice (19–21 weeks old) and TRPA1-/- mice (21–28 weeks old)	–	Acrolein (537 ppm, 8 times/4 weeks, inhalation) increases heart rate variability and myocardial desynchrony in B6129 mice but not in TRPA1-/- mice	Influence the autonomic nervous system	Thompson et al., 2019
	Female C57BL/6 and TRPA1-/- mice (15–30 weeks old)	–	TRPA1 knockout decreases acrolein-induced (3 ppm, 3 h) heart rate variability and arrhythmias	Cardiac autonomic function	Kurhanewicz et al., 2017, 2018
	Male spontaneously hypertensive rats (18–20 weeks old)	–	HC-030031 (5 mg/kg, i.p.) reduces diesel exhaust (32 ppm, 4 h)–induced and aconitine (1.5 mg/kg, i.p.)–induced ventricular arrhythmias	Restrains the activity of sympathetic and autonomic imbalance	Hazari et al., 2011
	Male Sprague–Dawley rats (15 weeks old)	–	AITC (30 mM) inhalation causes bradycardia atrioventricular blockade and prolonged PR intervals	Activates the vagus nerve	Hooper et al., 2016

### The Structure and Function of TRPA1

**FIGURE 1:**
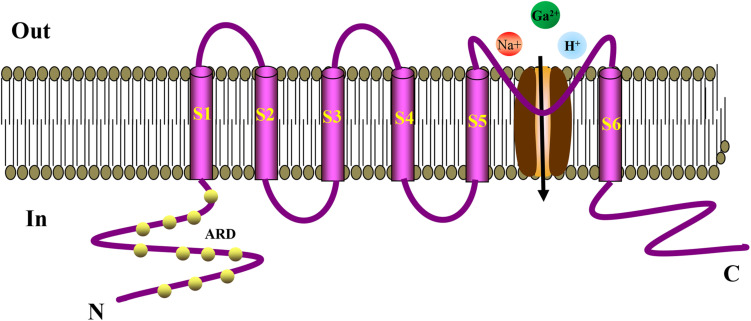
Schematic diagram showing the structure of TRPA1 channel. The TRPA1 channel has a six-time transmembrane structure (S1–S6), and there is a minute opening between S5 and S6 for ions to enter and exit the cell membrane. The N-terminus and C-terminus of the channel protein are located inside the cell. The N-terminus has a large number of characteristic ankyrin repeats, and the cysteine residues in these repeats serve as binding targets for the covalent modification of TRPA1 agonists. TRPA1 can increase the influx of extracellular cations, such as Na^+^, H^+^, especially Ca^2+^, thereby mediating a series of physiological reactions. ARD, Ankyrin Repeat Domain.

TRP ion channel is a non-selective cation channel located on the cell membrane, which is essentially a tetrameric form of Ca^2+^ influx channel (Hardie and Minke, 1992; Hofmann et al., 2017). Minke et al. discovered TRP in visual cells of *Drosophila* for the first time when studying the *Drosophila* experiments that is related to visual conduction (Cosens and Manning, 1969). It was observed that TRP only induces transient Ca^2+^ influx after light stimulation, generating a transient potential (Kumar et al., 2015). The letter “A” in the TPRA subfamily represents ankyrin, which has only one member, TRPA1. Like other TRP channels, ankyrin is embedded in the cell membrane and has a six-time transmembrane structure. They have a stoma area between S5 and S6 for ions to enter and exit the cell membrane. The N terminus and C terminus of the channel protein are located inside the cell (Schaefer, 2005; MacPherson, 2007). TRPA1 has similar characteristics of ankyrin repeats at its N terminus, whereas other TRP subfamily ankyrin repeats have only three to four times (Bodkin and Brain, 2011; Benemei et al., 2013). Ankyrin repeats are 33-amino-acid motifs that regulate the interaction between proteins. The cysteine residues in these repeats act as TRPA1 agonists covalently modifying the binding target (Dietrich et al., 2017). After TRPA1 activation, extracellular cations, such as Na^+^ and H^+^, are increased, especially Ca^2+^ influx, thereby mediating a series of physiological responses (Story et al., 2003). The structure of TRPA1 is shown in Figure 1. TRPA1 is best known as a sensor for environmental irritants giving rise to somatosensory modalities, such as pain, cold, itch, and other protective responses. TRPA1 plays an vital role in the pathological process of cough, oxidative stress, and inflammation (Chen et al., 2013; Benemei et al., 2014; Kurganov et al., 2014; Nassini et al., 2014; Winter et al., 2017). Recent studies have found that TRPA1 activation articulates in pancreatic islets, gastrointestinal tract, heart, and blood vessels (Pozsgai et al., 2010; Kun et al., 2014). Activation of TRPA1 in islet β cells can stimulate insulin secretion (Cao et al., 2012), in vascular cells it can improve endothelium-dependent diastolic function (Earley et al., 2009; Inoue et al., 2009), whereas in the intestine it can regulate the secretion of hunger hormones, GLP-1, and has effects on weight loss and regulating glucose and lipid metabolism (Derbenev and Zsombok, 2016). These studies concluded that TRPA1 might serve as a potential target to protect blood vessels and regulate metabolism.

TRPA1 is a non-selective cation channel and has the function of a receptor. TRPA1 channel activation includes multiple pathways. TRPA1 can be activated by a series of chemical stimuli, including cinnamaldehyde (Bandell et al., 2004), allicin (Bautista et al., 2005), allyl isothiocyanate (AITC) (Bandell et al., 2004; Jordt et al., 2004), ligustilide (Zhong et al., 2011), acrolein (Bautista et al., 2006), and nicotine (Andre et al., 2008; Talavera et al., 2009). In addition to exogenous agonists, recent studies found out that endogenous compounds released during tissue damage and oxidative stress can also activate TRPA1 channels, such as nitric oxide (NO) and hydrogen sulfide (H_2_S) (Andersson et al., 2008; Eberhardt et al., 2014), hydrogen peroxide (H_2_O_2_), 4-hydroxynon-enal (4-HNE), 4-oxoquinone (4-ONE), 4-hydroxyhexenal (4-HHE), and 15-deoxy-delta (12, 14)-prostaglandin J (2) (MacPherson et al., 2007b; Trevisani et al., 2007; Taylor-Clark et al., 2008). Besides, some synthetic compounds have also been reported, including ASP-7663 and Optovin (Kokel et al., 2013; Kojima et al., 2014). Low temperature is thought to activate the TRPA1 channel (Winter et al., 2017). However, TRPA1 is heat sensitive in snakes, lizards, and frogs (Kurganov et al., 2014). However, some agonists have a dual regulatory effect on TRPA1. For example, menthol activates TRPA1 at low concentrations and inhibits TRPA1 at high concentrations (MacPherson et al., 2006; Karashima et al., 2007; Alpizar et al., 2013). Table 2 lists commonly used TRPA1 agonists.

**TABLE 2:** TRPA1 agonists.

**Agonists**	**Source**	**EC_50_**	**References**
Allyl isothiocyanate (AITC)	Mustard	33 μM (mice)	Bandell et al., 2004; Jordt et al., 2004
		11 ± 1 μM (rat)	
Cinnamaldehyde (CA)	Cinnamon	100 μM (mice)	Bandell et al., 2004
Ligustilide	*Angelica sinensis*	44 μM (mouse)	Zhong et al., 2011
Allicin	Garlic	1.9 μM (human)	Jordt et al., 2004
		1.3 μM (mice)	Bautista et al., 2005
Nicotine	Tobacco	10 μM (mice)	Talavera et al., 2009
Hydrogen peroxide (H_2_O_2_)	Oxidative stress	230 μM (mice)	Andersson et al., 2008
4-Hydroxynon-enal (4-HNE)	Oxidative stress	10–27 μM (mice)	MacPherson et al., 2007b; Andersson et al., 2008; Taylor-Clark et al., 2008
4-Oxonon-enal (4-ONE)	Oxidative stress	1.5–1.9 μM (mice)	Andersson et al., 2008; Taylor-Clark et al., 2008
4-Hydroxyhexenal (4-HHE)	Oxidative stress	39–50 μM (mice)	Andersson et al., 2008; Taylor-Clark et al., 2008
15-Deoxy-delta(12,14)-prostaglandin J(2) ([15d-PGJ(2)])	Oxidative stress	5.6 μM (mice)	Andersson et al., 2008
ASP-7663	Synthetic	0.51 μM (human)	Kokel et al., 2013
		0.50 μM (mice)	
		0.54 μM (rat)	
Optovin	Synthetic	2 μM (mice)	Kojima et al., 2014

TRPA1’s exogenous antagonists are mainly complex organic or inorganic compounds. Previous studies have found some TRPA1 antagonists, such as ruthenium red, gentamicin, camphor, and high concentration menthol, but these antagonists also have regulatory effects on other certain ion channels (such as TRPV1, TRPV2); therefore, it is not specific. At present, the specific antagonists of TRPA1 mainly include xanthine derivatives, such as HC030031 and its derivatives (McNamara et al., 2007), TCS-5861528 (Wei et al., 2009), and GRC-17536 (Mukhopadhyay et al., 2014); and oxime derivatives, such as A-967079 (Chen et al., 2011) and AP-18 (Petrus et al., 2007; Defalco et al., 2010). The discovery of TRPA1 channel-specific antagonists has essential value for exploring the therapeutic uses of TRPA1. Therefore, researchers devote to finding TRPA1 channel-specific antagonists. Table 3 lists commonly used TRPA1 inhibitors.

**TABLE 3:** TRPA1 antagonists.

**Agonists**	**Structures**	**IC_50_**	**References**
HC-030031	Xanthine derivative	6.2 μM (human) 7.6 μM (rat)	McNamara et al., 2007
GRC-17536	Xanthine derivative	Not reported	Mukhopadhyay et al., 2014
TCS-5861528	Xanthine derivative	14.3 μM (human)	Wei et al., 2009
AP-18	Oxime	3.1 μM (human) 8.8 μM (rat) 4.5 μM (mice)	Petrus et al., 2007; Defalco et al., 2010
A-967079	Oxime	0.067 μM (human) 0.289 μM (rat)	Chen et al., 2011

## TRPA1 in the Cardiovascular System

### TRPA1 and Vascular Physiology

The contraction and relaxation of blood vessels form blood pressure, which provides impetus for the blood flow. Vascular smooth muscle regulates the tension of blood vessels under the joint control of vasoconstrictor nerve fibers and vasomotor nerve fibers, which in turn affects changes in blood pressure and blood flow. Long-term abnormal blood pressure is a key factor that causes cardiovascular disease (Earley, 2012). Vascular endothelial cells and vascular smooth muscle cells play an essential role in maintaining normal vascular physiology. *K*_Ca_ channels in endothelial cells and *K*_IR_ channels in cerebral arterial muscle cells are jointly mediated by vasodilation signals (Earley et al., 2009; Kohler and Ruth, 2010; Brunt et al., 2013). In addition, calcitonin gene–related peptides (CGRPs) secreted by vascular endothelial cells contribute to vasodilation (Aubdool et al., 2016).

In recent years, studies have demonstrated that TRPA1 has been explored in the vascular system and it was discovered that it has a vital role in the regulation of vascular tone (Earley et al., 2010; Aubdool et al., 2014). Formaldehyde activates the sensitive TRPA1 channel, causing the endothelium-dependent mechanism of Ca^2+^ influx and mediating the relaxation of isolated rat superior mesenteric artery. However, the same effect has not been found in the aorta (Jin et al., 2019). 4-ONE leads to obvious vasodilation, but this effect is not reflected in TRPA1-/- mice (Graepel et al., 2011). Both AITC and cinnamaldehyde significantly increased blood flow in the skin of anesthetized wild-type mice, but had no significant effect on TRPA1 knockout mice (Pozsgai et al., 2010). In cerebral circulation, the TRPA1 channel occurs in endothelial cells, and it is concentrated in the junction site of the endothelium muscle. Activation of the TRPA1 channel causes Ca^2+^ influx, mediating smooth muscle cell hyperpolarization and endothelium-dependent vasodilation (Sullivan et al., 2015). A study pointed out that stimulation of the TRPA1 channel causes vasodilation in a graded cerebral artery which is also caused by endothelial cell Ca^2+^ signaling (Qian et al., 2013). One study revealed that TRPA1 inhibitors and endothelial destruction antagonize AITC-induced cerebral artery vasodilation. AITC-induced arterial dilatation was blocked by treatment with small and medium-conductivity Ca^2+^-activated K^+^ channel blockers. Inwardly rectifying potassium channel treatment by blockers also blocked AITC-mediated vasodilation (Earley et al., 2009). The research indicated that Ca^2+^ influx via endothelial TRPA1 channels mediates cerebral artery vasodilation, which involves endothelial cell Ca^2+^-activated K^+^ channels and smooth muscle inward rectification K^+^ channels leading to hyperpolarization of cell membranes.

Other studies showed that the TRPA1 channel present in the peripheral vascular nerve mediates the chemical agonist through the mechanism of CGRP release, thereby mediating peripheral arterial vasodilation (Peixoto-Neves et al., 2019). The exogenous agonist of TRPA1 stimulates the release of CGRP and increases cerebral blood flow; this effect is blocked by TRPA1 and CGRP receptor antagonists (Kunkler et al., 2011; Aubdool et al., 2016).

The effect of TRPA1 activity on vascular physiology is also reflected in the regulation of blood pressure (Pazienza et al., 2014). Systemic administration of TRPA1 agonists will cause a transient hypotensive response, and then the heart rate and blood pressure continue to increase as a result of increased sympathetic nerve activity. Cinnamaldehyde relaxes the mesenteric artery in mice in a TRPA1-dependent manner (Pozsgai et al., 2010); however, the effect of intravenous administration of cinnamaldehyde on blood pressure is more complicated. The low dose of cinnamaldehyde causes the mesenteric artery to relax, which in turn causes a hypotensive response. The high dose of cinnamaldehyde causes a boosting response, which may involve the regulatory mechanism of the vagus nerve. Studies have indicated that vasodilation and antihypertensive effects are mediated by TRPA1 and TRPV1 (Ma et al., 2019). Vasodilation often uses propofol as an anesthetic, which in turn causes a hypotensive effect (Sinharoy et al., 2017). Currently, researchers knocked out the genes of TRPA1 and TRPV1 in mice, and the antihypertensive effect of propofol was significantly weakened (Sinha et al., 2015). However, after knocking out the mouse TRPV1 gene alone, the antihypertensive effect of propofol was not affected. Therefore, the TRPA1 ion channel, which mediates propofol-induced vasodilation, had little significance on TRPV1 ion channel. TRPA1 is a cold-stress cation channel that is activated at low temperatures (<17°C). In low-temperature environments, skin blood vessels contract, and vasodilation occurs after continuous contraction. TRPA1 plays a vital role in changes in vascular tone mediated by low temperatures (Aubdool et al., 2014).

**FIGURE 2:**
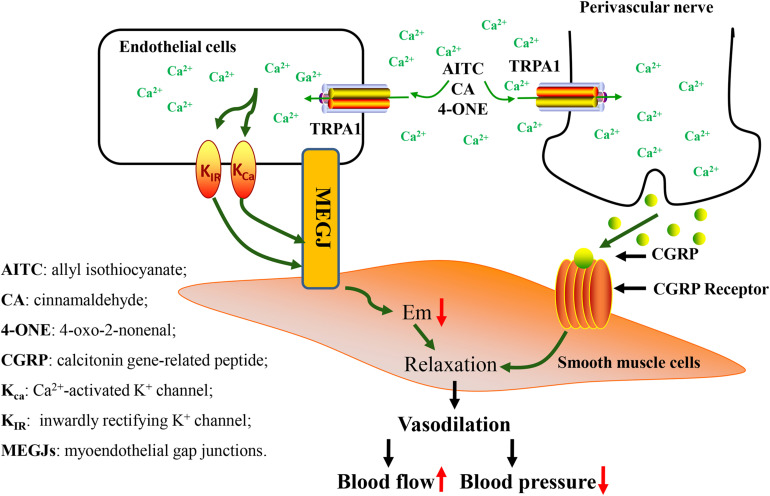
Vascular physiological changes caused by activation of TRPA1 channel. Activation of the TRPA1 channel causes Ca^2+^ influx, leading to hyperpolarization of endothelial cell membranes. This change in potential causes hyperpolarization of smooth muscle cell membranes, causing relaxation of muscle cells. In addition, activation of the TRPA1 channel induces an increase in [Ca^2+^], which leads to the release of the neuropeptide CGRP from perivascular nerve, thereby mediating vasodilation. Vasodilation causes changes in blood flow and blood pressure.

Therefore, TRPA1 causes vasodilation under the action of an agonist and increases blood flow, which in turn regulates blood pressure. TRPA1 channel regulates blood pressure in a bidirectional manner, which is related to the dose of the agonist. In addition, the activation of the TRPA1 channel under low-temperature environment causes changes in the tension of blood vessels (Figure 2). In conclusion, TRPA1 activity affects vascular function, but the exact role and significance of this channel in the cardiovascular system is yet to be determined.

## TRPA1 and Common Cardiovascular Diseases

### TRPA1 and Atherosclerosis

Atherosclerosis is one of the main reasons that lead to the causes of various cardiovascular diseases. The key initial factor of atherosclerosis is the accumulation of cholesterol (Yu et al., 2016), which in turn causes the formation of atherosclerotic plaques. This plaque leads macrophages to trigger a chronic inflammatory response. Macrophages clear excess peripheral cholesterol and convert the intracellular cholesterol into high-density lipoprotein (HDL) for excretion or storage. Macrophages play an essential role in the immune response of atherosclerosis, and the excessive intake of cholesterol into foam cells is the main reason for the formation of atherosclerosis (Lin et al., 2015). The outflow of cholesterol derived from oxidized low-density lipoprotein (oxLDL) is destroyed, and then the lipid accumulates in macrophages. When the lipid is overloaded and exceeds the metabolic capacity of macrophages, a large amount of lipids accumulate in the macrophages, which promotes the growth of blood vessel intima and the formation of necrotic nuclei, increasing the risk of plaque rupture. Furthermore, macrophages transform into foam cells, which further activates the inflammatory response and exacerbates the risk factors of plaque formation.

Cholesterol accumulation in macrophages determines lipid phagocytosis and cholesterol efflux. When the cholesterol content in the macrophages is too high, the macrophages will start the cholesterol efflux system and expel cholesterol from the cells to synthesize HDL. The main factors involved in cholesterol efflux are ATP binding cassette subfamily A member 1 (ABCA1) and ABCG1. Oxidative modified LDL causes lipid peroxidation of macrophages, which reduces the amount of HDL binding and causes cholesterol outflow disorder. TRPA1 expression in macrophage foam cells in the atherosclerotic aorta of apolipoprotein E-deficient [apoE (-/-)] mice was increased. Administration of the TRPA1 channel antagonist HC030031 or knockout of the TRPA1 [TRPA1 (-/-)] gene in apoE (-/-) mice can exacerbate atherosclerotic lesions, hyperlipidemia, and systemic inflammation (Zhao et al., 2016). Moreover, treatment with allyl isothiocyanate (AITC, TRPA1 agonist) can inhibit the progression of atherosclerosis in apoE (-/-) mice, but the TRPA1 (-/-) gene knockout atherosclerosis has no effect on sclerotic mice. Mouse macrophages showed that TRPA1 channels were activated by oxidized low-density lipoprotein (oxLDL). TRPA1 antagonists or knocking out the TRPA1 gene will exacerbate OxLDL-induced lipid accumulation in macrophages. Whereas inhibiting TRPA1 activity damages cholesterol efflux by downregulating the ATP binding cassette transporter, it does not change the internalization of oxLDL. Furthermore, macrophages activated by AITC weakens the inflammatory response induced by tumor necrosis factor-α, hence a recent study also reached a similar conclusion, TRPA1-/-ApoE-/- mice atherosclerotic plaque increased significantly (Wang Q. et al., 2020). The activation of TRPA1 by cinnamaldehyde significantly slows the progression of atherosclerosis. The researchers found out that inhibition of TRPA1 significantly stimulated the expression of M1 marker genes. TRPA1, which is upregulated in atherosclerotic plaques, can regulate the inflammatory phenotype of macrophages, thereby regulating the progress of atherosclerosis (Wang Q. et al., 2020). Therefore, TRPA1 plays a crucial role in the mechanism of atherosclerosis formation and it could be a therapeutic target for atherosclerosis and other metabolic diseases.

The current study confirmed that TRPA1 is associated with atherosclerosis, and it plays a key role in the macrophage cholesterol outflow and inflammation. However, it is not clear how the TRPA1 channel depends on the Ca^2+^ influx to mediate macrophage-derived foam cell formation. Lysophosphatidylcholine (LPC) is the main atherosclerotic lipid that stimulates the increase in mitochondrial reactive oxygen species (mtROS) and releases cytokines upon activation of the inflammatory body. A recent study examined the functional expression of TRPA1 in macrophages derived from the human acute monocytic leukemia cell line (THP-1). LPC induces the activation of THP-1-derived macrophages through Ca^2+^ influx, and TRPA1 inhibitors inhibit this activation. TRPA1 is involved in regulating Ca^2+^ influx caused by LPC. This result indicates that TRPA1 has important physiological functions in macrophages and is widely involved in the inflammation caused by LPC (Tian et al., 2020). This may be the early stage of macrophage foam cell. Further studies should be conducted to reveal the specific role of TRPA1 in the formation of macrophage foam cells.

### TRPA1 and Myocardial Fibrosis

Myocardial fibrosis refers to the excessive deposition of collagen fibers in myocardial tissue, the imbalance of various types of collagen, and the disorder of arrangement. Abnormal activation of cardiac fibroblasts is closely related to the formation of myocardial fibrosis. Myocardial fibroblasts are directed to the injury site under the influence of various pathological factors such as pressure overload, endothelial injury, hypoxic–ischemic injury, and generate a large number of extracellular matrix (ECM) mediated by local inflammatory factors, cytokines, and neurohumoral factors (Ma et al., 2012). Changes in the composition of myocardial ECM are the pathological basis of myocardial fibrosis. The process of myocardial fibrosis causes abnormalities in myocardial function, metabolism, and transmission, which leads to heart failure, various arrhythmias, and other heart disorders. Studies have shown that endogenous bioactive substances produced by cardiac fibroblasts regulate their activation. Furthermore, Ca^2+^-dependent signaling is essential for the proliferation of cardiac fibroblasts. Thus, understanding the effects and regulatory mechanisms of endogenous bioactive substances and Ca^2+^-dependent signaling on cardiac fibroblasts, which will provide new targets for anti-fibrosis strategies.

The autocrine of calcitonin gene-related peptide (CGRP) in myocardial fibroblasts may cause a positive cardiovascular effect, which relates to the inhibition of myocardial fibrosis (Russell et al., 2014). TRPA1 has been proven to promote the synthesis and secretion of CGRP and intracellular Ca^2+^ under the action of non-specific agonist AITC, while TRPA1 specific antagonist HC030031 can reverse these effects. CGRP is abundant in rat, mouse, and human myocardium, and it shows that cardiac CGRP develops from cardiac fibroblasts. In an animal experiment, calcitonin/CGRP knockout (KO) mice showed significant cardiac phenotypes, including cardiac remodeling and myocardial fibrosis (Supowit et al., 2005). The TGF-β superfamily is an important multifunctional growth factor that not only mediates cell growth, proliferation, and differentiation but also plays an important role in wound healing and the formation of ECM. It was discovered that TGF-β1 induced the activation of cardiac fibroblasts, and found that TGF-β1 can increase the mRNA expression of cardiac fibroblasts and the secretion level of CGRP. CGRP8-37 (CGRP receptor antagonist) exacerbates TGF-β1-induced cardiac fibroblast proliferation, differentiation, collagen production, and inflammation, thus indicating the decisive role of CGRP secretion in the formation of myocardial fibrosis. CGRP of cardiac fibroblasts is also an endogenous inhibitor of cardiac fibrosis. Furthermore, monocrotaline (MCT) is used to induce pulmonary hypertension, which gradually led to right ventricular fibrosis. The non-toxic TRPA1 agonist cinnamaldehyde (CA) inhibits MCT-induced pulmonary hypertension, which increases the RVSP, RV/LV + S, and right ventricular collagen accumulation, and downregulation of CGRP (Li et al., 2019). This indicates that TRPA1 activation has potential benefits in inhibiting TGF-β1-induced myocardial fibrosis.

Methylglyoxal (MG), also known as pyruvaldehyde, can activate TRPA1, which is a toxic by-product of glycolysis. It is distributed in all cells of mammals and is associated with various vascular diseases (Wang et al., 2019). In an *in vitro* study, myocardial fibroblast differentiation showed that it closely relates to MG-induced Ca^2+^ influx, and this effect was attenuated with the inhibition of TRPA1 channels, and the study suggests that MG-induced cardiac fibroblasts require TRPA1-mediated Ca^2+^-dependent signaling pathway (Oguri et al., 2014). In addition, inhibition of TRPA1 activity can significantly improve the degree of cardiac hypertrophy and myocardial fibrosis in mice, which may be related to weight loss and increase in aortic cross-sectional area (Wang et al., 2018).

Autocrine CGRP and Ca^2+^-dependent signaling of cardiac fibroblasts play an important role in the process of myocardial fibrosis formation. TRPA1 promotes the secretion of CGRP under the action of agonists and then inhibit the formation of myocardial fibrosis. However, TRPA1 inhibition antagonizes MG-induced Ca^2+^ influx. Subsequently, TRPA1 has opposite roles in the two pathways. At least it is clear that TRPA1 plays an important role in the pathological process of myocardial fibrosis. Further research should be done to consider the role of TRPA1 in different stages of myocardial fibrosis formation and use this potential therapeutic target more accurately.

### TRPA1 and Heart Failure

Heart failure is the ultimate destination of most cardiovascular diseases, which leads to heart circulation disorder caused by systolic or diastolic dysfunction. The activation of various neuroendocrine mechanisms such as the renin–angiotensin system, pro-inflammatory factors, and natriuretic peptide system closely relates to the development of heart failure (Geng et al., 2019; Hsu et al., 2020). Abnormal activation of the sympathetic nervous system is one of the most critical pathophysiological characteristics of heart failure (Sousa-Pinto et al., 2014). Excessive cardiac sympathetic reflex helps in nerve excitement. In an animal model experiment, capsaicin, as an agonist of TRPV1, enhanced cardiac sympathetic reflex. Capsaicin given after cutting off the vagus nerve in a rat heart failure model increased mean arterial pressure, heart rate, and renal sympathetic nerve activity (Adam et al., 2019). This suggests that TRPV1 activation enhances the degree of heart failure. Because of the similarity of TRPV1 and TRPA1 channels, TRPA1 activation may mediate cardiac sympathetic excitation. In addition, Ca^2+^ plays an important role in the myocardial excitation–contraction coupling mechanism, and myocardial cells in heart failure models show abnormal Ca^2+^ flow (Bers, 2006; Roe et al., 2015). The exact mechanism is unclear, however, as to the causes of abnormal sympathetic nerve excitement and heart muscle cell Ca^2+^ insufficiency. In the future, research must focus on these important targets for reducing heart failure.

The TRPA1 channel-mediated sympathetic reflex is unclear. It is worth noting that excessive sympathetic nerve excitation is a characteristic of chronic heart failure (CHF). In a recent study, the use of TRPA1 agonists to the surface of the heart and lungs increased heart rate and sympathetic nerve output in the control group of rats, on average arterial pressure, which changes in two phases. However, these effects are attenuated in CHF rats, thereby reducing the possibility of TRPA1 channel sensitization to CHF cardiopulmonary afferent (Adam et al., 2019). This shows that activation of the TRPA1 channel does not mediate an increase in sympathetic nerve reflexes and slightly decreases in CHF rats. In a mouse model experiment, the AITC stimulation of TRPA1 caused a dose-dependent increase in the peak [Ca^2+^]_I_ of isolated cardiomyocytes, time, and speed of reaching the peak of [Ca^2+^]_I_. However, no change in [Ca^2+^]_I_ was observed in cardiomyocytes pretreated with TRPA1 antagonist HC-030031 or cardiomyocytes obtained from TRPA1-/- mice (Andrei et al., 2017). TRPA1 stimulation leads to rapid phosphorylation of Ca^2+^/calmodulin-dependent kinase II (CaMKII). The stimulation of TRPA1 ion channels in cardiomyocytes mediates the activation of CaMKII-dependent signaling pathways, which increases cardiomyocytes contractile function (Marks, 2013; Roe et al., 2015). This may suggest that TRPA1 is involved in the regulation of heart failure through a Ca^2+^-dependent mechanism.

To our knowledge from previous studies, it seems that there is no direct evidence that TRPA1 activation causes sympathetic nerve excitation and that mediates heart failure. The current research results indicate that activation of the TRPA1 channel does not cause an increase in sympathetic reflex under heart failure. However, other studies suggest that the activation of TRPA1 channel helps to enhance cardiomyocyte contractility.

### TRPA1 and Arrhythmia

Arrhythmia is caused by the abnormal sinus node activation or excitement which occurs outside the sinus node, resulting in abnormal heartbeat frequency or rhythm. It is an important group of cardiovascular diseases, which includes premature atrial complexes, premature ventricular complexes, atrial fibrillation, and ventricular tachycardia (Goudis et al., 2015). Genetic factors and other external factors can cause arrhythmia. Numerous studies have shown that there is a potential connection between air pollution and arrhythmia (Watkins et al., 2013; Monrad et al., 2017). In addition, the heart rhythm regulated by the autonomic nervous system (ANS) and air pollution increase the possibility of arrhythmia by interfering with the ANS balance (Wu et al., 2015).

ANS plays an important role in regulating arrhythmia. Some studies suggest that PM_2_._5_ interferes with the balance of ANS, thus resulting in increased heart rate, reduced heart rate variability, and increased risk of arrhythmia (Watkins et al., 2013; Shen and Zipes, 2014; Feng et al., 2019). In another study, exposure to automobile exhaust increased sympathetic excitability in hypertensive rats, and this sensitivity decreased with the application of TRPA1 inhibitors (Hazari et al., 2011). On the other hand, AITC increased the incidence of arrhythmia in rats, and this effect is inhibited by cholinergic antagonists (Hooper et al., 2016). Furthermore, acrolein, a smoke component, increases the heart rate variability and arrhythmia in mice, and this effect disappears after knocking out the TRPA1 gene or giving TRPA1 inhibitors (Kurhanewicz et al., 2017, 2018; Thompson et al., 2019). Continued exposure to air pollution, toxic chemicals, and particulate matter (PM) causes inflammation of the lungs, leading to cough, asthma, chronic obstructive pulmonary disease (COPD), and other diseases. Studies have indicated that PM activates TRPA1 activity in the respiratory tract, which induces human lung disease (Deering-Rice et al., 2011; Akopian et al., 2016). As the level of systemic inflammation increases, the risk of atrial fibrillation in COPD subjects increases (Grymonprez et al., 2019), and there is a negative relationship between the level of asthma control and the increased risk of atrial fibrillation (Cepelis et al., 2018). This seems to suggest that TRPA1 activation indirectly causes arrhythmia. These findings indicate that the TRPA1 channel may stimulate arrhythmia through an airway inflammation and ANS imbalance.

The ANS can be divided into the sympathetic nervous system and the parasympathetic nervous system. The autonomic nervous system mainly innervates the muscles and smooth muscles of the internal organs, glands, and blood vessel walls. Pain, fear, and temperature can cause stress regulation of ANS. Owing to different types of harmful components in air pollution, the effect of TRPA1 activation is not absolute, however, activation of the sympathetic nervous system or parasympathetic nervous system is different (Middlekauff et al., 2014; Perez et al., 2015), thus, the role of the TRPA1 channel on cardiac autonomic nerves needs further investigation. Furthermore, previous reports stated that the activation of TRPA1 mediates the production of airway inflammation, causing lung inflammation, which increases the risk of arrhythmia to some extent. The activation of TRPA1 indirectly promotes the occurrence of arrhythmia.

### Relationship Between TRPA1 and Other Cardiovascular Disease Risk Factors

In addition to the primary physiological and pathological changes of the cardiovascular system, the occurrence and development of cardiovascular diseases are often secondary to other risk factors. There are many risk factors for cardiovascular disease, including diabetes, hypertension, dyslipidemia, age, genetics, and lifestyle habits (Park et al., 2020; Ungar et al., 2020). TRPA1 closely relates to many risk factors of cardiovascular disease.

### TRPA1 and Glycolipid Metabolism

Glycolipid metabolism disorder is a key factor in the formation of cardiovascular disease (Delitala et al., 2017). Metabolic diseases such as obesity and diabetes are important conditions for causing the development of cardiovascular diseases (Jokinen, 2015; Hansen, 2018). Diabetes manifests as insulin resistance and impaired insulin signaling. Hyperinsulinemia and hyperglycemia together accelerate the development of atherosclerosis (Bornfeldt and Tabas, 2011; Tabas, 2017). In particular, acute hyperglycemia weakens endothelial function and reduces the bioavailability of nitric oxide (Williams et al., 1998), increasing leukocyte adhesion (Perkins et al., 2015). In the state of hyperglycemia, the phenotype of vascular smooth muscle cells changes from a static, contracted state to an activated, proliferative state, which accelerates the progress of atherosclerosis (Zheng et al., 2007).

Atherosclerosis is the basis of other cardiovascular diseases; hence, many cardiovascular diseases develop from atherosclerosis (Frostegard, 2013). TRPA1 plays an important role in regulating glycolipid metabolism, which has important significance for the treatment of atherosclerosis (Zhao et al., 2016). Islet function, insulin level, and sensitivity are important factors that affect glucose metabolism. Studies have found that TRPA1 is abundant in rat islet β cells. AITC, H_2_O_2_, and 15d-PGJ2 activate TRPA1 in islet β cells and stimulate insulin secretion (Cao et al., 2012; Numazawa et al., 2012). Ghrelin, GLP-1, and other intestinal hormones affect the metabolism of glycolipids through various mechanisms. TRPA1 was present in endocrine cells in the mouse small intestine, and activating TRPA1 on these cells promoted GLP-1 secretion in a calcium-dependent manner (Shrestha et al., 2018). Cinnamaldehyde significantly inhibits the secretion of ghrelin by activating TRPA1 in the intestine to reduce food intake, and has the effect of weight loss and blood sugar reduction (Emery et al., 2015).

Cinnamaldehyde is a specific agonist of TRPA1 and has a moderate hypoglycemic effect on patients with type 2 diabetes (Rafehi et al., 2012). It has been confirmed that cinnamaldehyde promotes insulin and GLP-1 secretion (Numazawa et al., 2012; Shrestha et al., 2018), improves insulin sensitivity, and reduces liver fat deposition (Sartorius et al., 2014). The activation of pancreatic β-cell TRPA1 also promotes insulin secretion (Cao et al., 2012), as well as mesenteric adipose tissue which suggests that TRPA1 has a regulatory role in glucose metabolism. Intake of cinnamaldehyde has been shown to reduce visceral adipose tissue in high-fat and high-sugar fed mice (Tamura et al., 2012). This suggests that TRPA1 also has a potential regulatory role in lipid metabolism. In conclusion, the activation of TRPA1 indirectly reduces the incidence of cardiovascular diseases caused by glucose and lipid metabolism disorders.

### TRPA1 and Oxidative Stress

Oxidative stress refers to the tissue damage caused by the excessive production of highly reactive molecular oxygen species (ROS) and reactive nitrogen species (RNS) or the inability to eliminate them in the body (Del Rio, 2015). Although the organism has established a relatively complete anti-oxidative stress system during evolution, the balance between oxidation and anti-oxidation will eventually be broken under the joint action of various risk factors. The products of oxidative stress lead to cardiovascular metabolic diseases (Sverdlov et al., 2016; Faria and Persaud, 2017; van der Pol et al., 2019). ROS, RNS, 4-hydroxynon-enal (4-HNE), H_2_O_2_, NO, H_2_S, prostaglandin J2 (15d-PGJ2) are all TRPA1 direct or indirect agonists (Hinman et al., 2006; Taylor-Clark et al., 2009). This shows that oxidative stress metabolites can activate TRPA1, which is considered to be the endogenous substance that regulates TRPA1 in the body (Cao et al., 2012; Tamura et al., 2012). However, the relevant pathophysiological significance is currently unknown.

Previous studies confirmed that several TRPA1 agonists such as cinnamic aldehyde, artemisinol, and mustard oil have significant antioxidant activity, which activates the classic Nrf2/ARE antioxidant stress pathway (Huang et al., 2011; Zheng et al., 2011; Hsu et al., 2013). TRPA1 agonist cinnamaldehyde significantly antagonizes high glucose-mediated vascular endothelial oxidative stress through the Nrf2 pathway (Wang et al., 2015; Takahashi et al., 2018). Therefore, the role of TRPA1 and its agonists in the process of oxidative stress is worthy of further research. Among all subgroups of the TRP family, TRPA1 is the most sensitive to ROS. Its activation depends on the modification of the cysteine residue at the N-terminal end of the protein. Oxidation and peroxidation of cysteine residues cause residues to bind to disulfide bonds, which enhances TRPA1 activity (MacPherson et al., 2007a). TRPA1 is present in cardiomyocytes and is important in regulating myocardial reperfusion injury (Lu et al., 2016). A study showed that TRPA1 gene-knockout myocardial ischemia–reperfusion mice alleviated myocardial injury, indicating that TRPA1 channel activation mediates myocardial ischemia–reperfusion injury (Conklin et al., 2019). This may be related to the accumulation of free radicals and unsaturated aldehydes generated after myocardial ischemia and reperfusion to activate TRPA1 channel. The activated TRPA1 channel mediates a large amount of Ca^2+^ influx, aggravating inflammation and ischemia–reperfusion injury. Therefore, TRPA1 could be a potential drug target for reducing myocardial ischemia–reperfusion injury. In conclusion, the mechanism in which oxidative stress products activate TRPA1-mediated cardiovascular disease needs to be further discussed.

### TRPA1 and Aging

With aging, the damage of molecules, cells, and tissues in the body leads to an imbalanced state, which makes the body’s functions degenerate or cause diseases (Wu et al., 2019b). As a nutrient delivery channel of the human body, blood vessels play an important role in maintaining normal physiological functions. After entering old age, vascular aging becomes an independent risk factor for cardiovascular diseases (Costantino et al., 2016; He, 2016).

Current research reflects that mammalian lifespan is closely related to mitochondrial aging, oxidative stress, diet, insulin/IGF-1 signaling pathway, and UCP2 (Katic and Kahn, 2005; Duangjan et al., 2019), but the exact mechanism is still unclear. DAF-16 gene is a kind of lifespan regulator, called forkhead transcription factors of the O class (FOXO) in mammals (Jahn et al., 2020). Other studies revealed that hypothermia-activated TRPA1 regulates the downstream DAF-16/FOXO pathway through calcium-sensitive protein kinase C2 (PKC-2) signaling to extend lifespan (Xiao et al., 2013). However, low-temperature activation of TRPA1 failed to extend the lifespan of young individuals, which suggests that activation of TRPA1 plays a more important role in extending lifespan in adult and elderly individuals (Zhang et al., 2015). This is relatively related to the stable redox system in young individuals. These studies confirmed that low-temperature activation of TRPA1 could prolong the lifespan, but the specific mechanism still needs further clarification.

Aging is closely related to vascular aging; therefore, activating TRPA1 can significantly prolong life. Moreover, current research denotes that TRPA1 may have proper metabolic regulation and cardiovascular protection. Therefore, the role and mechanism of TRPA1 on age-related vascular damage should be explored further in combination with *in vitro* vascular culture and vascular endothelial-specific knockout mice.

## Conclusion

TRPA1’s role in cardiovascular diseases has received extensive attention, and it is directly or indirectly involved in the occurrence and development of cardiovascular diseases. This review describes the role of TRPA1 channels in common cardiovascular diseases. The TRPA1 channel is involved in mediating vascular physiological functions, which closely relates to the regulation of vasodilation and blood pressure. However, this regulation of blood pressure is biphasic. The activation of TRPA1 channel has a protective effect on the development of atherosclerosis; its channel blockade is beneficial for arrhythmia progression. The role of TRPA1 in the formation of heart failure and myocardial fibrosis is difficult to determine, and further experimental proof is needed. This review found evidence that TRPA1 channels relate closely to glucose and lipid metabolism, oxidative stress, and vascular aging. Considering the relationship between TRPA1 channel and various cardiovascular diseases, it may be a potential target for clinical treatment of cardiovascular diseases. In addition, according to the new drug design stage, the dual function of the TRPA1 channel should be fully considered.

## Author Contributions

SG and KK conceived the review and drafted the manuscript. SG and SZ revised the manuscript critically for important intellectual content. TL, PC, and RW provided funding for research. All authors approved the final version of the manuscript submitted.

## Conflict of Interest

The authors declare that the research was conducted in the absence of any commercial or financial relationships that could be construed as a potential conflict of interest.
